# Stress shielding in stemmed reverse shoulder arthroplasty: an updated review

**DOI:** 10.1051/sicotj/2024029

**Published:** 2024-09-20

**Authors:** Angelo V. Vasiliadis, Vasileios Giovanoulis, Nikolaos Lepidas, Ioannis Bampis, Elvire Servien, Sebastien Lustig, Stanislas Gunst

**Affiliations:** 1 Department of Orthopaedic Surgery, St. Luke’s Hospital Panorama-Thessaloniki 55236 Greece; 2 Orthopaedics Surgery and Sports Medicine Department, FIFA Medical Center of Excellence, Croix-Rousse Hospital, Lyon University Hospital Lyon 69004 France; 3 Ramsay Santé, Hôpital Privé Jean Mermoz, Centre Orthopédique Santy 24 avenue Paul Santy Lyon 69008 France; 4 Orthopaedics Department, Bioclinic 11524 Athens Greece; 5 Interuniversity Laboratory of Biology of Mobility, LIBM, EA 7424, Claude Bernard Lyon 1 University Lyon 69008 France; 6 Univ Lyon, Claude Bernard Lyon 1 University, IFSTTAR, LBMC UMR_T9406 Lyon 69622 France

**Keywords:** Reverse shoulder arthroplasty, Stress shielding, Risk factors, Clinical outcomes, Radiographic outcomes

## Abstract

*Background*: Reverse shoulder arthroplasty (RSA) is popular for the treatment of degenerative glenohumeral joint disease. Bone remodeling around the humeral stem related to stress shielding (SS) has been described. This review focuses on the specific radiological characteristics, risk factors, and clinical consequences of SS in RSA. *Methods*: A meticulous review was conducted of articles published between 2013 and 2023. Data on the definition, risk factors, and clinical impact of stress shielding were recorded. *Results*: Twenty-eight studies describing 2691 patients who had undergone RSA were included. The mean age of patients ranged from 63 to 80 years with mean follow-up periods of 12 months to 9.6 years. The prevalence of SS reached up to 39% at a 2-year follow-up. Females and elderly are typically at higher risk due to osteopenia. SS was more frequent with the use of long stems(>100 mm) compared to short stems(<100 mm). Stem design, onlay or inlay, and neck-shaft-angle did not influence SS. Frontal misalignment and a high filling ratio are riskfactors for SS. Biological factors also contribute to SS, associated with scapular notching. No correlation was found between SS and clinical outcomes. *Conclusions*: SS is common in patients with cementless implants after RSA, especially in female and elderly patients. It can be limited by implanting stems with a low diaphyseal filling-ratio, in correct coronal alignment. Risk factors for polyethylene debris, primarily scapular notching, should be avoided. The authors found no clinical consequences of stress shielding, but longer-term follow-up studies are required to confirm these findings.

## Introduction

Reverse shoulder arthroplasty (RSA) represents a successful treatment option for degenerative disease of the glenohumeral joint [1, 2]. It was initially proposed by Grammont for a rotator cuff disease and good clinical results have been achieved in mid- and long-term follow-up [3]. Many factors may contribute to alterations around the humeral stem, which is responsible for the load transfer to the bone, and therefore humeral stem design could lead to different biological reactions of the components [4]. Nagels et al. [5] defined SS as a reduction in bone density in the humerus, following Wolff’s law, due to a decrease in mechanical stress caused using humeral stem [6]. Additionally, Melis et al. [6] defined SS as one or more signs of bone narrowing or formation of osteolysis by measuring changes in the proximal humerus’s bone mineral density (BMD) and cortical thickness.

Preservation of bone has therefore become a major goal. Through the last decades, an important evolution regarding the humeral stem has been achieved. A progressive shift from long cemented stems to the adoption of long uncemented and more recently short cementless stems provides orthopedics surgeons with a variety of options [7]. However, there are several risk factors that we must consider, which may lead to bone resorption and implant loosening [8]. The degree of SS observed may vary depending on the type of implant used, the location of the implant, and the type of fixation [6, 8]. Therefore, careful monitoring of patients who have undergone RSA is important to detect any signs of SS and to address them early to avoid potential complications that may require revision surgery.

The present study is a review of all published articles describing SS in RSA to gather as much data as possible for the analysis of the risk factors and radiological and functional outcomes of SS, to best inform patient management.

## Methods

### Study design

A literature review was conducted across MEDLINE/PubMed from conception up to March 2023. Alone or in combination the terms “reverse shoulder arthroplasty”, “stress shielding”, “risk factors”, “clinical outcomes”, “functional outcomes” and “radiological outcomes” were used for data extraction. References listed in included articles were also analyzed to identify additional cases.

### Study selection

Articles were screened by title, abstract, and full text by two researchers (VG and AVV) working independently. Disagreements about inclusion were settled by a third investigator (SG) if no consensus could be reached. Excluded studies were listed.

The same authors (VG, AVV.) reviewed the papers separately and extracted the data for each included study. They used a predefined Microsoft Excel spreadsheet for data extraction. The following data were extracted: (1) study type details: authors, publication year, country, study design, level of evidence; (2) study population: sample size, age, level of evidence, gender, indications, follow-up; (3) range of motion; (4) functional/clinical outcomes; (5) radiographic outcomes (6) post-operative complications, revisions for any reason; (7) potential risk factors for SS.

### Selection criteria

The abstracts of these papers were reviewed to determine if eligibility criteria were met, which were: (i) patients undergoing RSA for any reason; (ii) full-text articles in indexed journals; (iii) articles published in English; (iv) human studies; (v) mean follow-up of at least 12 months. Exclusion criteria were: (i) treatment using total shoulder arthroplasty (TSA) and hemiarthroplasty, (ii) studies included only stemless prosthesis, (iii) availability of only an abstract, (iv) review article and meta-analysis, (v) case reports and (vi) engineering and mathematical modeling studies. The risk of bias was assessed using the Cochrane Risk of Bias Tool for randomized studies and the Newcastle-Ottawa Scale for observational studies. This assessment was conducted by two independent reviewers (VG and AVV), with disagreements resolved by a third reviewer (SG).

## Results

**Figure 1:**
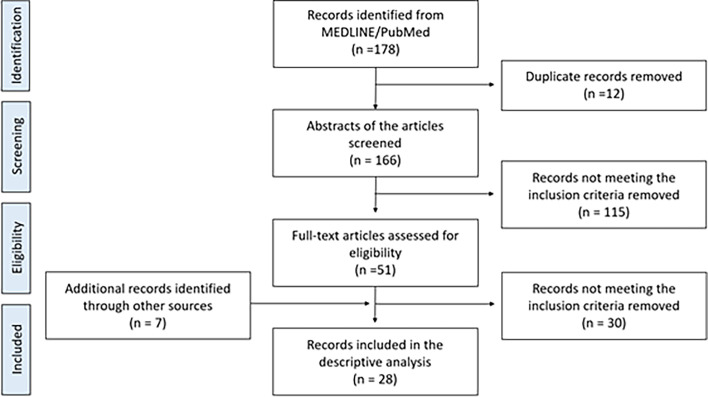
Flowchart of studies included.

Of the 161 articles initially identified, 28 articles were found eligible after the screening process for this review. A summary of the flowchart of the literature search can be illustrated in Figure 1. Two out of the 28 studies (7.1%) were prospective [9, 10] 25 (89.3%) were retrospective [4, 6, 11–33], and one case series [34]. Eight studies (28.6%) had a level of evidence IV [9, 15, 17, 20, 23–25, 31], whereas 20 studies (71.4%) had a level of evidence III (Table 1) [4, 6, 10–14, 16, 18, 19, 21, 22, 26–30, 36, 37]. All cohort studies were graded as good/high quality based on the Newcastle scale.

**Table 1:** The basic characteristics of the included studies and participants.

Study	Design/Level	Gender (M/F)	Age (years)	FU (months)	Indications
Yokoya et al. [35]	Retrospective/III	42/93	M:78.2/F:77.6	M:36.5/F:30.6	Primary
					RCA: 55.6%
					MRC tear: 31.9%
					Fractures: 6.7%
					OA/RA: 5.9
Nourissat et al. [36]	Retrospective/III	4/14	74.6	72	Primary
					MRC tear
					OA
Erickson et al. [37]	Retrospective/III	295/282	68.5 to 69.4	24	Primary
Giordano et al. [11]	Retrospective/III	10/66	76 to 76.2	36 to 46.8	Primary
					RCA
					OA
Kim et al. [12]	Retrospective/III	25/79	72.1 to 73.5	13.2 to 13.5	Primary
Kramer et al. [13]	Retrospective/III	8/43	74	27	Primary
					PHF
Lopiz et al. [14]	Retrospective/III	29/39	78 to 80	26.5 to 37.8	Primary
					PHF
Valenti et al. [15]	Retrospective/IV	9/15	68.7	44.7	Primary
					MRC tear
Mazaleyrat et al. [4]	Retrospective/III	29/103	74.5 to 75.3	9 years (108 m)	Primary
					RCA: 56.8%
					MRC tear: 36.3%
					OA: 12.8%
Nagase et al. [31]	Case series/IV	1/12	74	57.9	Primary
					RA: 100%
Polisetty et al. [16]	Retrospective/III	40/52	73.2 to 74.8	29 to 31	Primary
					MRC tear
					OA
Abdic et al. [17]	Retrospective/IV	42/80	74	–	Primary
					RCA: 63 to 78%
					OA: 33 to 41%
					RA: 4 to 5%
Brolin et al. [18]	Retrospective/III	31/89	70.6	35.2	Primary
Denard et al. [19]	Retrospective/III	49/70	69.3 to 71.1	36.1 to 36.8	Primary
					RCA
					FRC repair
					OA
Inoue et al. [20]	Retrospective/IV	23/58	76.5	18.5	Mixed
					RCA: 81.3%
					FRC repair: 8.3%
					Malunion PHF: 4.2%
					OA: 4.2%
					Revision: 2%
Aibinder et al. [21]	Retrospective/III	65 both	68	3.8 years	Primary
					RCA: 50.8%
					OA: 38.5%
					AVN: 3.1%
					Post-traumatic: 1.5%
					RA: 6.1%
Merolla et al. [22]	Retrospective/III	23/51	74.7 to 75.8	24	Primary
					RCA
Raiss et al. [23]	Retrospective/IV	77 both	72	28	Primary
					OA
Harmsen and Norris [24]	Retrospective/IV	82/82	68.2	36.6	Primary
					RCA: 42.1%
					MRC tear: 29.9%
					OA: 17.7%
					RA: 4.9%
					Infection: 1.8%
					PHF: 1.2%
Weber-Spickschen et al. [25]	Retrospective/IV	6/8	70	43	Revision
					Haematoma
					Infection
Al-Hadithy et al. [9]	Prospective/IV	8/29	79	5 years	Primary
					RCA
Wiater et al. [26]	Retrospective/III	35/66	71.95 to 72.47	32.4 to 37	Primary
					RCA
					MRC tear
Holschen et al. [27]	Retrospective/III	16/26	72 to 76	34 to 42	Primary
					RCA: 100%
Melis et al. [6]	Retrospective/III	20/45	69.4	9.6 years	Mixed
					RCA: 73.8%
					MRC tear: 13.8%
					Revisions: 12.4%
Mazaleyrat et al. [28]	Retrospective/III	24/80	74.6 to 75.1	9.5 years	Primary
					RCA: 53–54%
					MRC tear: 33–36%
					OA: 11–13%
Erickson et al. [29]	Retrospective/III	145/131	67.8 to 69.4	12 minimum	Primary
Giuseffi et al. [30]	Retrospective/III	15/29	76	27	Primary
					RCA: 75%
					AVN:13.6%
					OA: 4.5%
					RA: 6.8%
Schnetzke et al. [10]	Prospective/III	4/20	63	25	Primary
					OA: 62.1%
					Post-traumatic: 27.6%
					AVN: 6.9%
					RA: 3.4%

Abbreviations: M, male; F, female; FU, follow-up; RCA, rotator cuff arthropathy; MRC tear, massive rotator cuff tear; OA, osteoarthritis; RA, rheumatoid arthritis; PHF, proximal humeral fracture; FRC repair, failed rotator cuff repair; AVN, avascular necrosis.

In total, 2691 patients (1029 males and 1657 females from the available data) were included. The mean age ranged from 63 years [10] to 80 years [14]. Their mean follow-up period ranged from 1 year [29] to 9.6 years [6] (Table 1). One article (4%) only included cases of revision RSA [22], two papers (7.1%) considered cases of both primary and revision RSA, and the remaining 25 papers (89%) considered primary RSA patients only (Table 1). The most common aetiologies among patients who had undergone primary RSA with a known etiology were rotator cuff arthropathy (proportions ranging from 50.8 to 81.3%) [20, 21], glenohumeral osteoarthritis (4.2 to 41%) [17–20], massive rotator cuff tear (14 to 36.3%) [4, 6] and inflammatory arthropathy (3.4% to 6.8%) [10, 30]. In mixed studies of primary and revision RSA [6, 20], the main indications for revision RSA were glenoid loosening of an anatomic prosthesis, conversion for humeral head replacement; and failed hemiarthroplasty or TSA. The study of exclusively revision RSAs involved 15 patients who had undergone conversions from TSA to RSA because of hematoma or infection (Table 1) [25].

Overall, 22 studies examined the clinical/functional outcomes and these studies used 12 clinical/functional subjective scores (Table 2). The most commonly used score was the Constant score in 13 studies (59.1%) [6, 9–15, 22, 26, 27, 31, 37]. The visual analog scale (VAS) pain score [11, 12, 15, 16, 19, 22, 24–26, 29, 30, 35] and the American Shoulder and Elbow Surgeons (ASES) score [11, 12, 14, 16, 19, 21, 24–26, 29, 35, 37] were each used in 12/22 studies (55%). The subjective shoulder value (SSV) score was used in five studies (23%) [10, 15, 25–27] and the simple assessment numeric evaluation (SANE) score [19, 24, 29], the Western Ontario osteoarthritis of the shoulder (WOOS) score [25, 29, 35] and the Neer score [21, 29, 30] were mentioned in three studies (14%). The Simple Shoulder Test score [19, 22] and the Oxford [9, 25] shoulder score were used in two studies (9.1%), and the Japanese Orthopaedic Association (JOA) score [36], Shoulder36 (version 1.3) score [31] and the disability of arm, shoulder and hand (DASH) score [10] were used in one study each (Table 2).

**Table 2:** Detailed data on ROM, strength, functional/clinical outcomes, radiographic outcomes, and complications in RSA.

Study	ROM	Strength	Functional/Clinical outcomes	Radiographic outcomes	Complications
Yokoya et al. [35]	FF, IR, ER	–	JOA	AP, axillary, scapular Y	–
	Not improve		Not improve	Cortical thinning and osteopenia: 50.3%	
				Calcar osteolysis: 34.8%	
				Great tuberosity: 29.6%	
Nourissat et al. [36]	–	–	Constant	AP, lateral	Dislocation (5.5%)
			↓ in patients with resorption	Cortical contact: 79%	
			ASES	Humeral resorption: 21%	
Erickson et al. [37]	FF, IR, ER	–	ASES, WOOS, VAS	Grashey, axillary	Short-stem:
	Short stem: ↑FF, IR		Short stem had better scores	Short stem: ↑FR_met_, FR_dia_	Periprosthetic fracture (0.4%)
					Revision (0.3%)
					Standard-stem:
					Infection (0.8%)
					Revision (0.4%)
Erickson et al. [29]	FF, IR, ER	–	ASES, WOOS, SANE, Neer, VAS	Grashey, axillary	–
	More IR in short-stem		Less VAS, better ASES and WOOS in short-stem vs. standard-stem	Higher FR in standard-stem	
				Valgus alignment: 8.6% (standard-stem) vs. 2.2% (short-stem)	
				Calcar osteolysis: 12.9% (standard-stem)	
Giordano et al. [11]	FF, IR, ER, Abd	Handheld dynamometer	Constant, ASES, VAS	AP, IR, ER	Short-onlay-stem:
	Improve	↑post-op.	Improve	Scapular notcing: 35.2% (short stem) and 23.8 (long stem)	Loosening, glenoid (2.9%)
					Dislocation (2.9%)
					Long-inlay-stem:
					Infection (2.4%)
					Dislocation (2.4%)
Kim et al. [12]	FF, IR, ER	–	Constant, ASES, VAS	AP, axillary, lateral	Low FR (19.6%):
	High FR: ↑FF, IR		Improve	Low FR: ↓ stress shielding	Acromial fracture (3.9%)
			High FR: ↑ASES		Persistent pain (3.9%)
					ROM difficulty (11.8%)
					High FR (20.8%):
					Acromial fracture (3.8%)
					Persistent pain (%)
					ROM difficulty (9.8%)
Kramer et al. [13]	–	–	Constant	AP, axillary, Neer	Non-cemented:
			Improve	Great tuberosity healing: 71% (non-cemented), 79% (cemented)	Revision (5.9%)
				Lesser tuberosity healing: 82% (non-cemented), 94% (cemented)	Cemented:
				Scapular notching: 6% (non-cemented), 18% (cemented)	Revision (5.9%)
Lopiz et al. [14]	FF, IR, ER, Abd	–	Constant, ASES	AP, axillary	Non-cemented (8.6%)
	Cemented: ↑ FF (127° vs 108° in non-cemented)		Not differences cemented vs. non-cemented	Radiolucent lines: 17.8% (cemented), 8.3% (non-cemented)	Cemented (17.7%)
				Tuberosity healing: 64% (cemented), 91% (non-cemented)	
Valenti et al. [15]	FF, IR, ER, Abd	–	Constant, VAS, SSV	AP, axillary, IR, ER	–
	Improve		Improve	Radiolucent lines: only cemented	
				Tuberosity resorption: non-cemented	
				Stress shielding: non-cemented	
Mazaleyrat et al. [4]	–	–	–	AP, axillary	Non-cemented (8.5%)
				Tuberosity resorption: cemented > non-cemented	Cemented (4.2%)
Mazaleyrat et al. [28]	–	–	–	AP, axillary	Non-cemented (5.4%):
				Tuberosity resorption: 59% (non-cemented), 30% (cemented)	Periprosthetic fracture (3.6%)
				Stress shielding: 39% (non-cemented)	Humeral loosening (1.8%)
				Scapular notching: 41% (both non-cemented and cemented)	Cemented (1.8%):
					Humeral loosening (1.8%)
Nagase et al. [31]	FF, IR, ER, Abd	–	Constant, Shoulder36	Scapular Y	0%
	↑ FF, Abd		Improve	Stress shielding: 21.4%	
	↓ ER			No loosening	
				No heterotopic ossification	
Polisetty et al. [16]	FF, IR, ER	–	ASES, VAS, SST	AP, scapular Y	Inlay design:
	Onlay: greater FF and ER		No differences between inlay and onlay humeral design	Tuberosity and calcar resorption: 73.9% (onlay design)	Scapular notching (8.7%)
					Acromial fracture (8.7%)
					Onlay design:
					Scapular notching (8.7%)
					Acromial fracture (13.6%)
Abdic et al. [17]	–	–	–	AP	–
				Malaligned: larger stem and ↑ FR	
Brolin et al. [18]	–	–	–	AP	Cemented:
				Osteolysis: higher in cemented	Humeral loosening (1.7%)
				Stress shielding: higher in non-cemented	
Denard et al. [19]	FF, ER	–	ASES, VAS, SST, SANE	Grashey	Total (8.4%):
				High adaptive changes: 3.2% (non-cemented)	Infection (10%)
					Stiffness (10%)
					Instability (10%)
	Improve		Improve	Calcar osteolysis: 43% (non-cemented), 58% (cemented)	Scapular fracture (50%)
					Persistent pain (10%)
					Popping (10%)
					Revision (2.5%)
Inoue et al. [20]	–	–	–	AP	–
				↑ bone resorption in greater tuberosity, lateral diaphysis, calcar	
Aibinder et al. [21]	FF, IR, ER	–	ASES, Neer	AP	Total (9%) both TSA and RSA:
	Improve		↑ ASES	Stress shielding: 14%	Infection (33.3%)
			Excellent Neer (41%)	Calcar resorption: 23%	Fracture of humeral tray (22.3%)
				Scapular notching: 5%	Glenoid loosening (11.1%)
					Instability (33.3%)
Merolla et al. [22]	FF, IR, Abd	–	Constant, VAS	Grashey, axillary, scapular Y	Inlay design:
	Improve		↑ Constant	Glenoid radiolucency: inlay > onlay	Dislocation (5.6%)
			↓ VAS	Scapular notching: 39% (inlay), 5% (onlay)	Instability (2.8%)
				Humeral radiolucency: 25% (inlay), 10% (onlay)	Onlay design:
				Cortical thinning, spot weld and tuberosity resorption: inlay > onlay	Scapular fracture (5.3%)
					Acromial fracture (2.6%)
					Infection (7.9%)
					Revision (2.6%)
Raiss et al. [38]	–	–	–	AP, 3 different rotation views	Total (7.8%):
				FR influenced the radiographic changes	Infections (2.6%)
				Cortical contact led to high bone adaptations	Dislocation (1.3%)
				Bone adaptations: female > male	Acromial fracture (2.6%)
Harmsen and Norris [24]	FF, ER, Abd	Abd. strength in scapular plane improve (dynamometer)	ASES, VAS, SANE	AP, axillary, scapular Y	Total (15.1%):
	Improve		↑ ASES, SANE	Radiolucent lines (met): 97.4%	Acromial/scapular fracture (19.4%)
			↓ VAS	Cortical resorption: no	Deep infection (19.4%)
				Osteolysis: no	Dislocation (13.9%)
					Transient neuropathy (11.1%)
					Superficial infection (8.3%)
					Periprosthetic fracture (2.8%)
					Humeral shaft fracture (2.8%)
					Malposition (2.8%)
					Retained drill fragment (2.8%)
Weber-Spickschen et al. [25]	–	–	ASES, Oxford, WOOS, SSV, VAS	AP, axillary, scapular Y	Dislocation (7.1%)
			↑ ASES, Oxford, WOOS, SSV	Radiolucent lines: 0% (glenoid), 7.1% (humeral stem)	
			↓ VAS	Stress shielding: 14.3%	
				Resorption: no	
				Loosening: no	
Al-Hadithy et al. [9]	FF, IR, ER, Abd	–	Constant, Oxford	AP, axillary, lateral	Total (10.8%):
	Improve		↑ Constant, Oxford	Scapular notching: 68%	Glenoid implant failure (5.4%)
				Stress shielding: 10.8%	Acromial fracture (2.7%)
				Heterotrophic ossification: 42%	Broken glenoid screw (2.7%)
					Revision (2.7%)
Wiater et al. [26]	FF, IR, ER	–	Constant, ASES, SSV, VAS	AP, laterals	Non-cemented (7.8%):
	Improve		↑ Constant, ASES, SSV	Loosening: no	Systemic (3.1%)
			↓ VAS	Stress shielding: 7.8% (non-cemented)	Dislocation (4.7%)
					Cemented (16.2%):
					Systemic (8.1%)
					Dislocation (2.7%)
					Infection (2.7%)
					Acromial fracture (2.7%)
Holschen et al. [27]	FF, ER, Abd	–	Constant, SSV	AP, axillary	135° neck-shaft angle:
	Not differences between operated and non-operated side		No differences between 155° and 135° neck shaft angle	Scapular notching: 66% (155°) and 33% (135°)	Infection (4.8%)
				Calcification: 48% (155°) and 38% (135°)	
				Stress shielding: 29% (155°) and 10% (135°)	
Melis et al. [6]	FF, ER, ER (90°), Abd	–	Constant	AP, axillary	Total (10.3%):
	No differences between cemented and non-cemented		No differences between cemented and non-cemented	Scapular notching: 88%	Instability (5.9%)
			Very satisfied and satisfied: 84.5%	GT resorption: 69% (cemented) and 100% (non-cemented	Humeral fracture (2.9%)
				LT resorption: 45% (cemented) and 76% (non-cemented	Acromial fracture (1.5%)
					Cemented:
					Humeral loosening (11.8%)
					Non-cemented:
					Humeral loosening (5.9%)
Giuseffi et al. [30]	FF, ER	–	Neer, VAS	AP, scapular Y	Total (6.9%):
	Improve		Neer: Excellent (61.4%)	Malaligned: 4.6%	Brachial plexus abnormality (2.3%)
			↓ VAS	Heterotopic ossification: 41%	Dislocation (2.3%)
					Infection (2.3%)
Schnetzke et al. [10]	FF, ER, Abd	Arm strength (ISOBEX dynamometer)	Constant, SSV, DASH, Pain	AP, axillary, scapular Y	Total (8.3%):
	Improve		↑ Constant, SSV	Cortical thinning/osteopenia: 42.1%	Acromial fracture (8.3%)
			??? DASH	High adaptations: 10.5%	
			↓ Pain		

Abbreviations: ROM, range of motion; FF, forward flexion; IR, internal rotation; ER, external rotation; Abd, abduction; JOA, Japanese Orthopaedic Association; AP, anteroposterior; ASES, American Shoulder and Elbow Surgeons; WOOS, Western Ontario Osteoarthritis of the Shoulder; VAS, visual analogue score; FR_met_, filling ratio metaphyseal; FR_dia_, filling ratio diaphyseal; SSV, subjective shoulder value; SST, Simple Shoulder Test; SANE, Simple Assessment Numeric Evaluation; ADLEIR, Activities of daily living with requirement for external and internal rotation score; DASH, Disability of Arm, Shoulder and Hand; TSA, total shoulder arthroplasty; RSA, reverse shoulder arthroplasty; CVS, cerebrovascular stroke.

Postoperative range of motion (ROM) was evaluated in 19/28 studies (68%) (Table 2) [6, 9–12, 14–16, 19, 21, 22, 24, 26, 27, 29–31, 36, 37]. Mean ROM was measured with a goniometer in terms of forward flexion, abduction, external rotation, and internal rotation. Studies that compared preoperative and postoperative ROM found significant improvements at final follow-up [9–11, 15, 19, 21, 22, 24, 26, 27, 30]. Two studies found that patients with a short humeral stem had significantly higher ROM in active forward flexion and internal rotation compared with those with standard humeral stems [29, 35]. Two studies found that patients with cemented prostheses had greater ROM in forward flexion and abduction but lower ROM in external and internal rotation than those with uncemented implants [6, 14]. One study found that patients with an onlay humeral prosthesis had significantly greater ROM in active external rotation and forward flexion [16]. Finally, one study comparing outcomes in patients with different implant neck-shaft angles (155° vs. 135°) found no differences between the groups in any of the considered movements (forward flexion, abduction, and external rotation) [27].

Arm strength was only evaluated in three studies (11%), two of which investigated abduction strength in the scapular plane using a dynamometer [10, 24] while in the third study [11] strength measurements were performed with a handheld dynamometer in forward elevation, abduction, external rotation with the arm at the side, and external rotation with the arm at 90° abduction All three studies found significant improvements in mean arm strength postoperatively (Table 2).

All 28 studies mentioned radiographic outcomes, based on true anteroposterior, internal/external rotation, axillary, and scapular Y views. Scapular notching was evaluated in 15/28 studies (54%) [4, 6, 9–11, 13, 14, 16, 19, 26–28, 31, 36, 37] in each case using the Sirveaux classification [32]. Six studies [4, 13, 14, 18, 26, 28] compared complications between patients with cemented and uncemented prostheses (Table 2). Three studies [14, 18, 26] found more complications in the cemented group, two studies [4, 28] reported more complications in the non-cemented group, while one study found a similar rate of complications between cemented and non-cemented group [13]. Three studies [11, 16, 22] compared complication rates among patients with onlay and inlay implants, with the only design associated with more complications in each case. Two studies [11, 35] compared complication rates associated with short and long humeral stems, and both found no difference (Table 2). The most commonly reported risk factors for SS in RSA were the type of fixation (identified in 17/28 studies, 61%) [4, 11–15, 17–19, 23–26, 31, 35–37], the type and design of the humeral stem (in 14 studies, 50%) [4, 9, 11, 16, 17, 19–24, 35–37], the filling ratio (in eight studies, 29%) [12–14, 17, 23, 35–37], female sex (in three studies, 11%) [18, 20, 36] and stem alignment (in one study, 4%) [17] (Table 3).

**Table 3:** Detailed data on potential risk factors for stress shielding in RSA.

Study	Sample	Age (years)	Risk factor	Outcomes	FU (months)
Yokoya et al. [35]	135 shoulders	77.8	– Type of stem (length)	– Predictors of stress shielding: long stems, cementless stems, larger proximal FR and larger metaphysis diameters	34.6
			– Type of stem (thickness)	– FR_prox_ > 0.7 is an independent predictors of stress shielding	
			– Type of fixation	– Stress shielding: NSA 155° (26.9%), NSA 135° (59.6–93.5%)	
			– Filling ratio	– Stress shielding was seen in 50.4% of the shoulders	
			– Gender		
Nourissat et al. [36]	19 shoulders	74.6	– Filling ratio	– Low rate of stress shielding: press-fit ovoid stem of 100 mm length	72
			– Type of fixation	– No correlation between the filling ratio and resorption of the bone	
			– Type of stem (length)	– Lower rate of stress shielding: humeral neck-shaft angle of 145°	
Erickson et al. [37]	577 shoulders	Short-stem: 68.5	– Type of fixation	– No differences on stress shielding between short inlay press-fit stem and standard press-fit stem	Minimum 2 y
	Short-stem: 220	Standard-stem: 69.4	– Filling ratio	– There is an ideal FR_met_ around 60–70% to minimize stress shielding	
	Standard-stem: 357		– Type of stem (length)	– Short (60–65 mm) stems lead to comparable rate of stress shielding compared with standard-length (111–147 mm) stems	
			– Type of stem (design)		
Giordano et al. [11]	76 shoulders	Onlay: 76.2	– Type of fixation	– Higher rate stress shielding: onlay with 145° neck-shaft angle cohort	Onlay: 46.8
	Onlay: 34	Inlay: 76	– Type of stem (length)	– Lower rate of stress shielding: long-inlay straight stems	Inlay: 36
	Inlay: 42		– Type of stem (design)	– Lower rate of stress shielding: humeral neck-shaft angle of 155° (7.1%) versus 145° (17.6%)	
Kim et al. [12]	104 shoulders	HFR: 72.1	– Filling ratio	– Patients with low FR stems with autogenous bone grafting had significantly less humeral stress shielding than patients with high FR and press-fit stem	HFR: 13.5
	HFR: 53	LFR: 73.5	– Type of fixation		LFR: 13.2
	LFR: 51				
Kramer et al. [13]	51 shoulders	All: 74	– Type of fixation	– Not correlation of higher FR_dist_ with severe stress shielding	27
	Cemented: 34	Cemented: 74.4	– Filling ratio		
	Cementless: 17	Uncemented: 74.1			
Lopiz et al. [14]	68 shoulders	Cemented: 78	– Type of fixation	– Stress shielding: 30.4% of uncemented group	Cemented: 37.8
	Cemented: 45	Uncemented: 80	– Filling ratio	– Not correlation of FR_diaph_ with stress shielding	Uncemented: 26.5
	Cementless: 23				
Valenti et al. [15]	24 shoulders	68.7	– Type of fixation	– Signs of stress shielding appeared exclusively in non-cemented stems	44.7
	Cemented: 5				
	Cementless: 19				
Mazaleyrat et al. [4]	140 shoulders	Cemented: 74.5	– Type of fixation	– Proximal humeral osteolysis: inlay-type RSA with cementless stems	Cemented: 9.6 y
	Cemented: 70	Cementless: 75.3	– Type of stem (design)	– Proximal humeral osteolysis can in part be attributed to stress shielding	Uncemented: 8.9 y
	Cementless: 70			– Signs of stress shielding, such as osteopenia, was only in cementless (89%)	
Nagase et al. [31]	14 shoulders with rheumatoid arthritis	74	– Type of fixation	– Stress shielding: 21.4% of humeral cemented stems	57.9
Polisetty et al. [16]	92 shoulders	Onlay: 74.8	– Type of stem (design)	– Stress shielding, onlay-style had more instances of GT (73.9%) and calcar (39.1%) resorption	Onlay: 29
	Onlay: 46	Inlay: 73.2		– Inlay-style prosthesis: better prevention of	Inlay: 31
	Inlay: 46			humeral stress shielding	
Abdic et al. [17]	124 shoulders	74	– Type of fixation	– Small stems (≤90 mm) are implicated in a higher incidence of malpositioning with high endocortical contact	n/a
			– Filling ratio	– Smaller malaligned stem (≤90 mm): higher rate of endocortical contact distally, which associated with stress shielding	
			– Type of stem (length)	– Short stems (<94 mm) may decrease stress shielding	
			– Alignment of stem		
Brolin et al. [18]	120 shoulders	70.6	– Type of fixation	– Uncemented stems had significantly more internal stress shielding	35.2
	Cemented: 49		– Gender	– Neither age nor gender had a correlation with stress shielding	
	Cementless: 71				
Denard et al. [39]	119 shoulders	Cemented: 71.1	– Type of fixation	– Proximal stress shielding was more common with press-fit fixation	Press-fit: 36.1
	Cemented: 26	Cementless: 69.3	– Type of stem (length)	– Proximal lateral stress shielding: 25% in cemented and 68% press-fit group	Cemented: 36.8
	Cementless: 93			– Standard length stems (111–147 mm) can lead to stress shielding	
Inoue et al. [20]	48 shoulders	76.5	– Gender	– Female gender and onlay-type stem were significant independent risk factors for grade 4 bone resorption (GT, lateral diaphysis and calcar), due to stress shielding	18.5
			– Type of stem (design)	– The mechanism of bone resorption after RSA may be related to stress shielding and polyethylene wear	
Aibinder et al. [21]	100 shoulders	68.2	– Type of stem	– GT stress shielding: 18.5% in cementless short humeral component	3.8 y
	TSA: 35				
	RSA: 65				
Merolla et al. [22]	74 shoulders	Inlay: 75.8	– Type of stem (length)	– The standard stem (inlay-design) showed higher stress shielding and higher rate of GT resorption	Inlay: 35.1
	Aequalis II: 36	Onlay: 74.7	– Type of stem (design)	– Stress shielding: NSA 155° (cortical thinning, 26.9%; spot welds, 11%), NSA 145° (cortical thinning, 26%)	Onlay: 29.1
	Ascend flex: 38				
Raiss et al. [38]	GI: 150 TSA	GI: 68	– Filling ratio	– Stress shielding: higher in the uncemented group	GI: 32
	GII: 77 RSA	GII: 72	– Type of fixation	– Stress shielding: 35% of stems, with high bone adaptations in 17%	GII: 28
			– Type of stem (length)	– FR ≥ 0.8 increases the rate of stress shielding	
				– Stress shielding in 97% in RSA in stems with 66–94 mm of length	
Harmsen and Norris [24]	232 shoulders	68.2	– Type of fixation	– Internal stress shielding is observed to the coated diaphyseal press-fit humeral stem	26.4
			– Type of stem (length)	– Internal stress shielding of 97.4% in the early post-operative period	
Weber-Spickschen et al. [25]	15 shoulders	70	– Type of fixation	– Stress shielding: 13.3% in cementless humeral stem	43
	Cemented: 1				
	Cementless: 14				
Al-Hadithy et al. [9]	41 shoulders	79	– Type of stem	– Proximal humeral bone resorption: 10% in uncemented and hydroxyapatite coated humeral stem, due to stress shielding	5 y
Wiater et al. [26]	101 shoulders	Cemented: 71.95	– Type of fixation	– Stress shielding: 7.8% in uncemented stems	Cemented: 37
	Cemented: 37	Cementless: 72.47		– Stress shielding and bone resorption: laterally at the metadiaphyseal junction	Cementless: 32.4
	Cementless: 64				

Abbreviations: n/a, not applicable; FU, follow-up; y, years.

## Discussion

**Figure 2:**
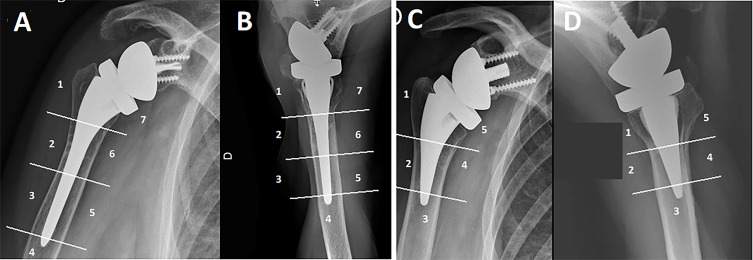
Humeral zones for standard-length and short humeral stems according to the Gruen classification [40] adapted to the shoulder are used to locate sites of osteolysis, radiolucent lines, and stress shielding. (A) Anteroposterior view and (B) Axillary view of standard-length stem (>100 mm). (C) Anteroposterior view and (D) Axillary view of short stem (<100 mm).

**Figure 3:**
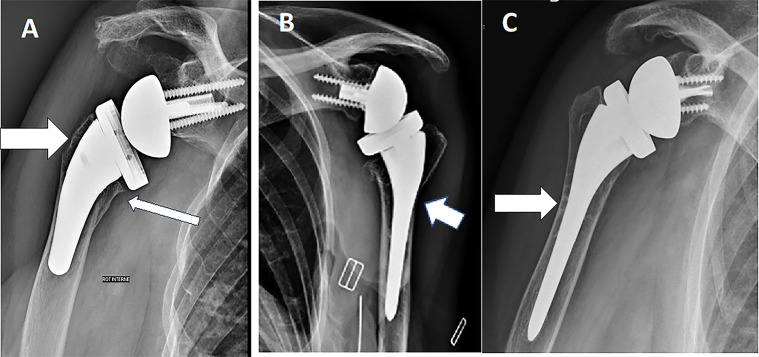
(A) Anteroposterior (AP) radiograph of a Right shoulder with evidence of under the baseplate osteolysis (thin arrow) and greater tuberosity osteolysis (thick arrow). (B) AP radiograph of a Left shoulder with evidence of lateral cortical thinning (arrow). (C) AP radiograph of a Left shoulder with evidence of spot welds (arrow).

The present review found that the prevalence of stress shielding at a minimum of 3 years [26, 31] in RSA with uncemented humeral stems was reported at between 7.8% and 39% [10, 18, 21, 26, 30, 31]. Numerous factors affect SS after RSA, while clinical outcomes were comparable between different fixation methods and implant designs [6, 24, 27]. Radiologic outcomes after RTSA usually assess the following signs: radiolucency, condensation lines, cortical thinning, spot weld, loosening, bone resorption (near calcar or tuberosities area), and subsidence for the humeral implant and scapular notching, bone spurs, heterotopic ossifications, radiolucency and loosening for the glenoid implant [11, 26, 37]. These features are evaluated on standardized radiographs and located in seven zones based on the Gruen classification [40] and adapted to the shoulder by Melis [6] or in five zones for short humeral stems (Fig. 2). An example of several X-rays based on the author’s clinical experience is illustrated in Figure 3. This review suggests that limiting the SS for bone preservation and potential revisions is desired which agrees with the current tendency in the recent literature [39]. This work also supports the use of implants with short stems and a low filling ratio (FR) have a protective role against stem subsidence.

Inoue et al. identified female sex as a risk factor for bone resorption induced by SS because of micro-architectural bone deterioration related to osteoporosis [20]. The high prevalence of osteoporosis in elderly female patients was also identified as a risk factor for SS by Morita et al. [33] and Yokoya et al. found that female sex was significantly associated with cortical thinning and osteopenia [36]. In contrast, Brolin et al. found no correlation between sex and SS (cortical thickness) [18] possibly because their patients were relatively young.

Several studies [4, 9, 13–15, 18, 19, 21, 23, 25, 26, 36] have reported high rates of SS in patients after uncemented RSA, while stress shielding in patients with cemented stems has only been evaluated in one study [31]. Mazaleyrat et al. [4] observed signs of SS, such as osteopenia, in up to 89% of cementless stems at a mean follow-up of 9 years. Kramer et al. [13] in a comparative retrospective study of cemented vs. uncemented RSA for proximal humerus fracture, found SS in all uncemented group patients (100%), compared to other studies with press-fit fixation of a primary RSA performed with standard-length stems [19] or uncemented RSA (18.5%) for degenerative cases [21]. Thus, this review claims that stress shielding may be a phenomenon specifically associated with cementless stems.

This study seems to prove that long stems with >100 mm in length can lead to SS in terms of cortical thinning and osteolysis. Three studies also identified diaphyseal fixation with long stems (>100 mm) as a risk factor for stress shielding in RSA [11, 22, 36]. Similarly, Harmsen and Norris [24] have observed a high rate of SS of 97.4% with the use of a diaphyseal press-fit humeral stem in RSA [11, 19, 22, 24]. Also, it has been shown that shorter stems (<94 mm) may reduce the risk of SS [17]. On the contrary, one recent work compared the use of short stems (60–65 mm) and long stems (111–147 mm) provides comparable findings in terms of SS [35]. The authors reported a possible explanation for the higher metaphyseal and diaphyseal filling ratio in the short stem group.

The relationship between stress shielding and the design of the humeral component (inlay vs onlay) has only been investigated in four studies. Inlay stems seem to be protective against humeral SS compared with onlay stems, which seem to be a risk factor for bone resorption in the greater tuberosity, lateral diaphysis, and calcar region [11, 16, 20]. However, Merolla et al. [22] found that inlay stems were associated with higher rates of cortical thinning and spot welds.

Stress shielding may also vary with the neck-shaft angle (NSA) of the prosthesis, with various studies [11, 18, 36, 37] reporting SS rates ranging from 7.1% for a long straight 155° NSA inlay stem [11] to 93.5% for a humeral component with a 135° NSA [36]. However, the rates of SS associated with different NSAs have only been compared in three studies [17, 20, 27]. Yokoya et al. [36] found that the prevalence of SS was significantly higher in patients with a 135° NSA prosthesis (59.6% to 93.5%) than in those with a 155° NSA stem (26.9%). Meanwhile, Giordano et al. [11], found a SS rate of just 7.1% in patients treated with a long straight inlay implant with a 155° NSA. On the contrary though, Merolla et al. [22] found that the use of a larger NSA (155° vs 145°) was associated with higher rates of SS in terms of cortical thinning (58%) and spot welds (11%). It is well known that correct coronal alignment of the humeral stem is crucial to avoid discrepancies with the true NSA of the humerus. These offsets, particularly with short stems, can lead to diaphyseal endocortical contact, a finding that is also related to SS [17]. At present, it is not clear what the ideal NSA is and surgeons must be weary of the use of curved cementless short stems because stem misalignment is associated with NSA alteration, endocortical contact, and increased SS [33].

Despite the high rates of SS associated with cementless humeral stems [4, 19], recent studies have found that this may be reduced thanks to specifically designed prostheses [17, 20, 37]. The influence of stem geometry on SS is difficult to assess because many factors may be involved, such as the NSA, inlay or onlay design, and humeral lateralization and distalization, and these characteristics are not compared directly in existing studies

In five studies, a high filling ratio was proposed as a possible risk factor for the development of stress shielding [12, 17, 23, 35, 36], while three studies did not observe a relation between FR and SS [13, 14, 37]. Based on this literature review, there may be an ideal metaphyseal and diaphyseal FR of <0.7, to minimize SS [23, 36, 37]. This correlates with the work by Kim et al. [12] who found that SS rates one year after RSA were much lower in patients with low filling ratios (≤0.64) and impaction grafting, than in those with high filling ratios (≥0.75). Similarly, Abdic et al. [17] found that smaller stem sizes (≤82 mm) were protective against SS because they ensured lower filling ratios. More recently, Yokoya et al. reported that a proximal filling ratio above 0.7 was an independent predictor of SS and proximal humeral osteolysis at a mean follow-up of 34 months [36]. However, the fact that three studies found no correlation between filling ratios and SS suggests that other factors may be implicated, although the small size of these studies may also have contributed to their negative findings [13, 14, 37].

Varus or valgus misalignment of the stem in the proximal humerus is another recognized risk factor for SS [17]. The risk of misalignment with shorter humeral prostheses has been explained by the inability to rely on diaphyseal alignment and misaligned short stems are most often implanted in valgus [17, 21]. The use of an uncemented curved short humeral implant may also increase the risk of SS because of the higher risk of distal endocortical contact [17]. Despite several studies showing favorable outcomes with short humeral stems compared with conventional long humeral stems, misalignment is more common with short stems [41]. Poor alignment may lead to excessively horizontal or vertical stems, with an increased risk of scapular notching and dislocation [34]. Some short stems have been designed with different lengths to avoid poor alignment, but further long-term comparative studies are required to determine the most suitable stem length in terms of alignment, endocortical contact, and SS [41]. Overall, however, existing results indicate that stress shielding can be limited by ensuring good frontal alignment and a low filling ratio. In a cadaveric study, Gunst et al. [42] showed that these objectives could be achieved with short stems by inserting them with standard-length compactors.

As previously documented in anatomic TSA, inflammation caused by polyethylene debris or metal degradation is also a risk factor for radiological changes around the implants inRSA. Peduzzi et al. [43] found that under-the-baseplate osteolysis could be related to the presence of polyethylene implants and polyethylene wear, as suggested previously by others [38, 44–46]. These debris have been identified as the cause of high-grade tuberosity resorption in patients with cementless stems [4]. Other works [9, 32] have found a significant association between scapular notching and tuberosity resorption. Stress shielding may therefore result from a combination of mechanical and biological factors.

Regarding clinical outcome, Mazaleyrat et al. [4] found no correlation between SS and clinical outcomes at the last follow-up. They did find although that patients with cemented stems had significantly better active ROM than those with press-fit stems. Melis et al. [6], comparing outcomes in patients with cemented versus uncemented stems, found that SS was more common in patients with uncemented stems but that clinical outcomes did not differ significantly between the two groups at up to 8 years, except internal rotation ROM, which was significantly higher in the uncemented group. Similarly, Denard et al. [19], reviewing 93 press-fit and 26 cemented stems with the same design at a minimum follow-up of 2 years, found no difference in clinical outcomes or ROM between the two groups despite SS being more common in the press-fit group. Finally, two studies investigating the utility of adjustable systems [24] or with different inclination angles of the humeral component [27] found no association between the presence of SS and clinical results. To date therefore, there are no data in the literature indicating any association between SS and clinical outcomes in RSA; however, further studies with longer follow-up are required to confirm this.

This review has several limitations. It includes RSA for all indications, including both primary and revision RSAs, which introduces additional variables that could complicate the analysis of the confounding effect of diagnosis on stress shielding. For instance, RSA performed for revision procedures or proximal humerus fractures cannot be directly compared to primary RSAs. Additionally, we could not perform subgroup analyses and the authors could only describe the risk factors. Due to the high heterogeneity of the study parameters, a meta-analysis was not feasible. Nevertheless, this is the first study to comprehensively summarize the risk factors for the development of stress shielding after RSA.

## Conclusions

Stress shielding is a common phenomenon after cementless RSA, particularly in female and elderly patients, due to osteoporosis. The risk of SS can be reduced by implanting stems in correct coronal alignment with a low diaphyseal filling ratio. Factors known to promote the release of polyethylene debris, notably scapular notching, should be avoided. While no clinical consequences of SS have yet been reported, this finding needs to be confirmed at longer follow-up.

## Data Availability

Data are available on request.
